# Supplementary figures and images for: The alteration of LBX1 expression is associated with changes in parameters related to energy metabolism in mice
Source: PLoS One. 2024 Aug 7;19(8):e0308445. doi: 10.1371/journal.pone.0308445 (PMC11305531; doi:10.1371/journal.pone.0308445)

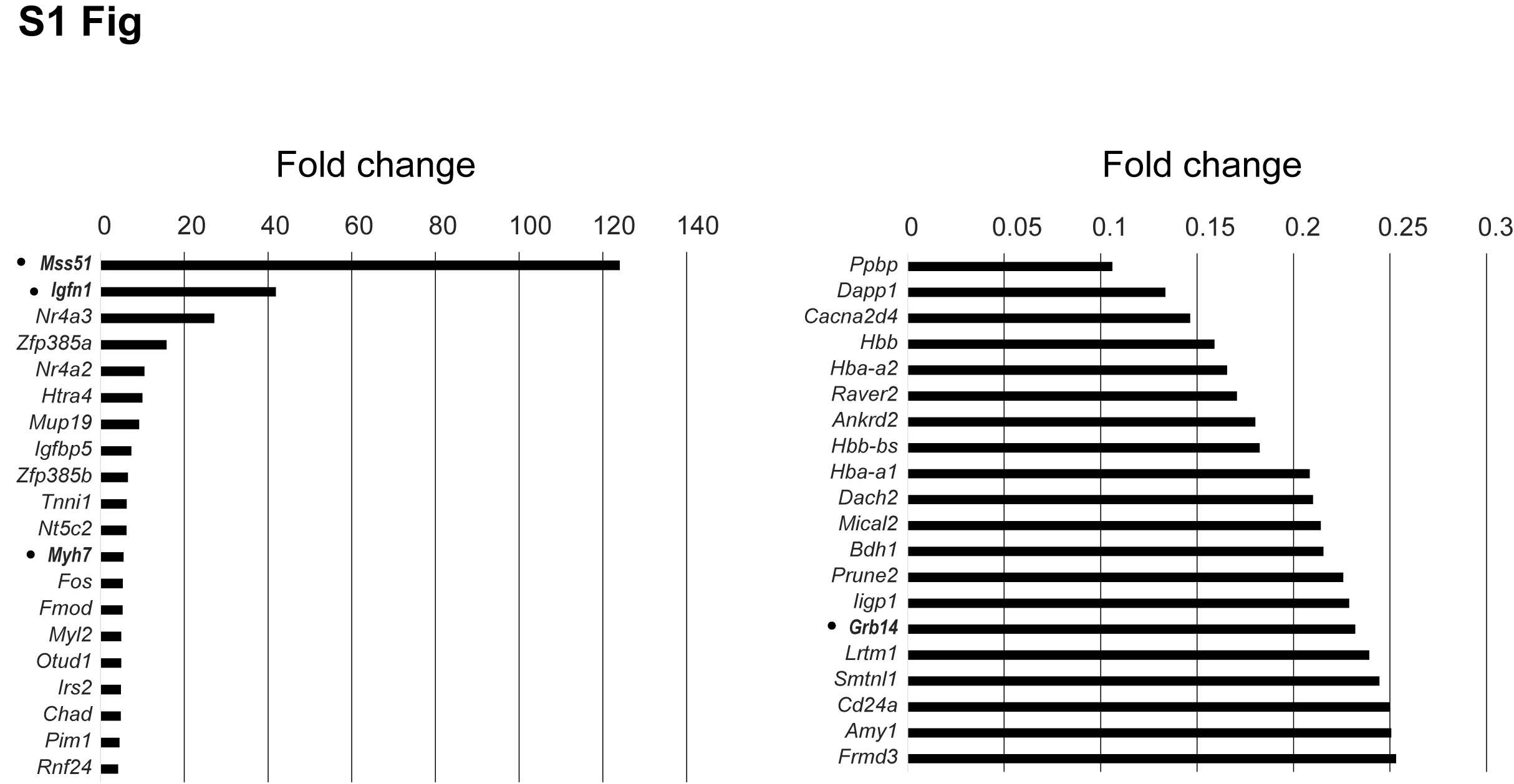

Supplement: S1 Fig — Lists of 20 genes found to be most up- (left panel) and down-regulated (right panel) in skeletal muscle tissue of Lbx1ΔMus mice compared to Ctrl mice by microarray analysis. Black dots indicate genes with a potential role in energy metabolism whose increased or decreased expression in skeletal muscle was validated by quantitative PCR. The transcriptional expression levels of Bdh1, Igfbp5, Irs2, Nr4a2, and Nr4a3 were also examined, but the difference in expression could not be reproduced by quantitative PCR. (TIF) [file pone.0308445.s001.tif]

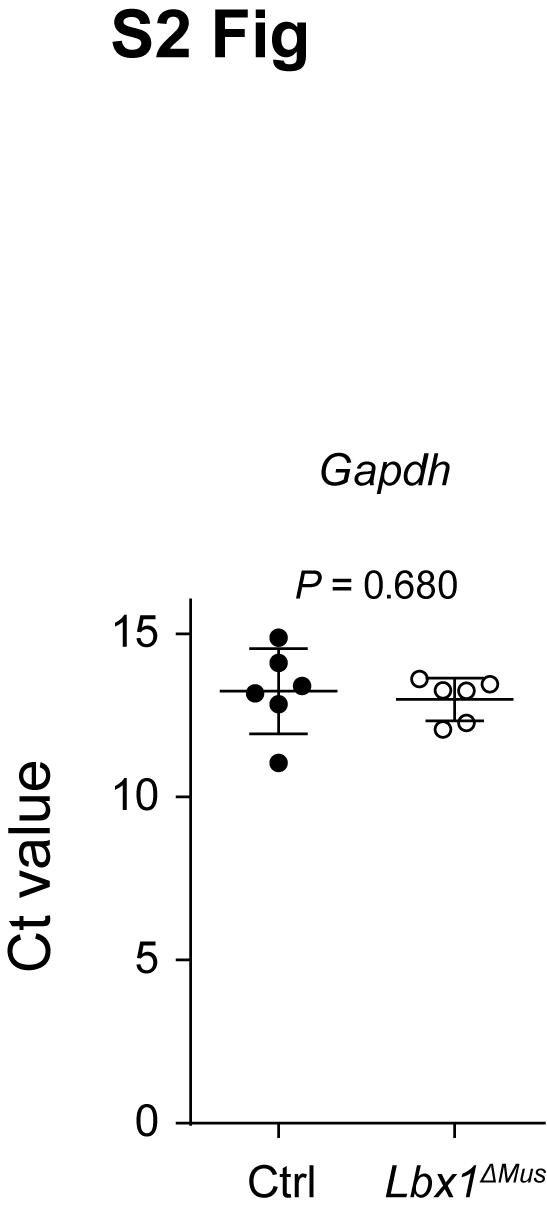

Supplement: S2 Fig — N = 6. Mann-Whitney U test. (TIF) [file pone.0308445.s002.tif]

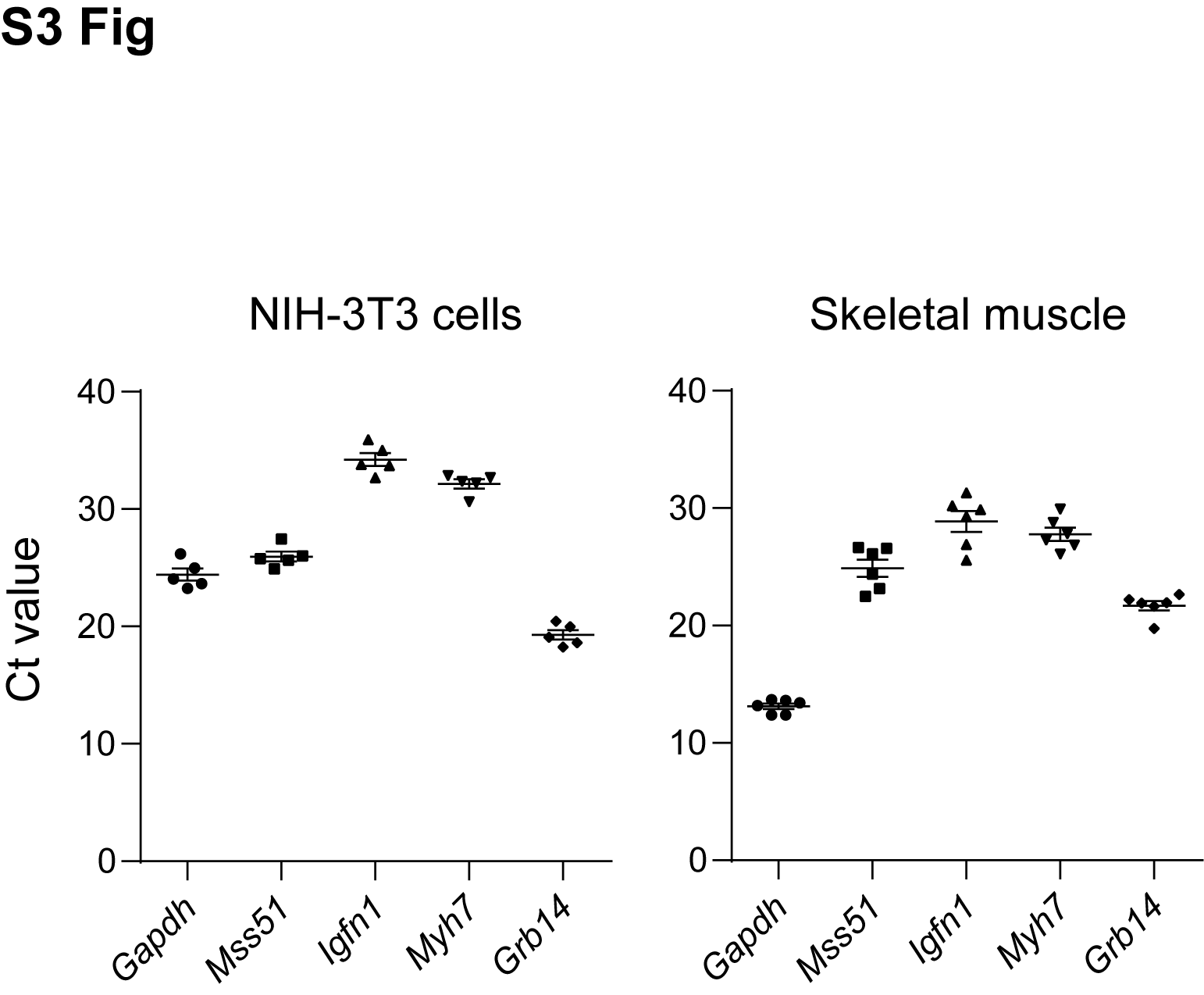

Supplement: S3 Fig — N = 6. (TIF) [file pone.0308445.s003.tif]

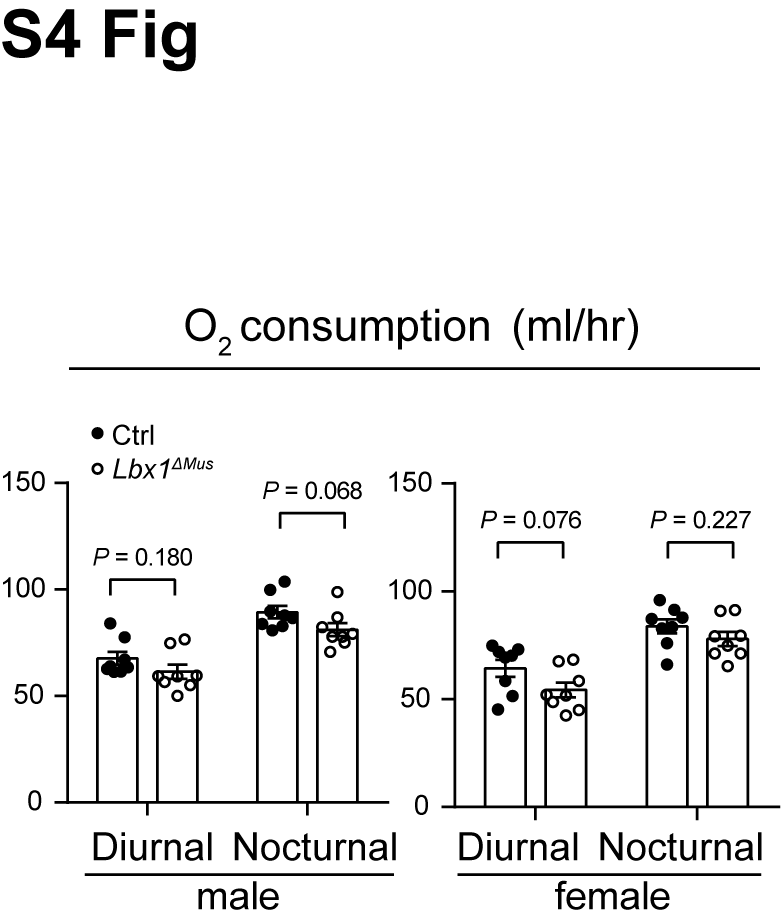

Supplement: S4 Fig — N = 6. (TIF) [file pone.0308445.s004.tif]

## S1 raw images

Fig 4B (boxed areas)

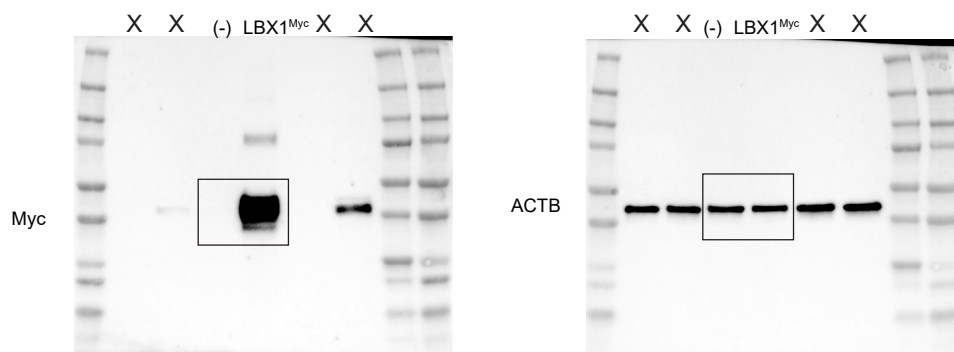

Supplement: S1 Raw images — (PDF) [file pone.0308445.s005.pdf]
