# Supplementary material for: The alteration of LBX1 expression is associated with changes in parameters related to energy metabolism in mice
Source: PLoS One. 2024 Aug 7;19(8):e0308445. doi: 10.1371/journal.pone.0308445 (PMC11305531; doi:10.1371/journal.pone.0308445)
Supplement: Attachment — Submitted filename: rebuttal letter.docx [file pone.0308445.s006.docx]

Point-by-point Responses

**First, we would like to thank the reviewers for their insightful and constructive critiques of the manuscript. By addressing the issues raised by the reviewers, we believe that our manuscript has substantially improved.**

Reviewer #1:

1. Lbx1 KO mouse are significantly smaller than the controls and the difference is believed to be mostly because of smaller fat mass. If the lean mass is really comparable between both groups, it is misleading to normalize any output to total body weight, as fat mass does not affect much parameters like blood volume or energy expenditure. If both strains are injected with the same dose of glucose, which seems more appropriate in this case, the differences in blood glucose and its AUC would be less pronounced. Similarly, energy expenditure should not be normalized to body weight (or unnormalized data should be present in parallel at least).

**Response:** **We agree with the reviewer that in some cases it would be preferable to perform GTT experiments using lean body mass rather than total body weight as a reference. However, in the present study, GTT experiments (and other experiments related to energy expenditure) were performed on 11-week-old mice, at which point there is little fat accumulation in Ctrl mice, and the difference in perigonadal fat volume between Ctrl and *LbxΔMus* mice is negligible (Fig 2A). Therefore, we believe that it would not be completely irrational to use total body weight as a reference in our experiments (the difference in body weight is likely due to the hypoplastic forelimbs, as we have previously described*). Nevertheless, we have included a graph showing basal O2 consumption with and without normalization with body weight (Fig 3E and S4 Fig: please also see the next response). When adjusted for body weight, O2 consumption was slightly higher in *LbxΔMus* mice than in Ctrl mice, but this difference was abolished when unadjusted. However, considering that total activity is significantly lower in *LbxΔMus* mice than in Ctrl mice (Fig. 3H) and there is no difference in food intake (Fig 3G), it is conceivable that overall energy expenditure is higher in *LbxΔMus* mice than in Ctrl mice.**

***Matsuhashi Y, et al. 2022. J Orthop Res 41:884-890**

2. While VO2max is useful parameter regarding muscle function and strength, basal O2 consumption and 24h O2 consumption would be more relevant to link the data to changes in body weight. Such energy expenditure can be directly compared to energy intake. Moreover, calculation of respiratory exchange ratio would reveal whether the KO mice consume more lipids, further supporting the link to reduced fat mass.

**Response: As requested by the reviewer, we have included figures showing basal O2 consumption and respiratory quotient (Figs 3E and 3F). We found that O2 consumption was comparable or slightly higher (the difference did not reach statistical significance) in *LbxΔMus* mice compared to Ctrl (especially during the nighttime), even though activity is lower in *LbxΔMus* mice compared to Ctrl (Fig 3H). On the other hand, we did not observe any difference in the respiratory quotient between Ctrl and *LbxΔMus* mice.**

3. The selection of cell line for the cell culture experiments need some justification. For comparison of cell culture experiments to in vivo model of muscle specific Lbx1 manipulation, it would make much more sense to use some muscle cell line.

**Response: We agree with the reviewer that cell culture experiments using NIH3T3 cells are not an ideal substitute for an in vivo model of muscle-specific *Lbx1* manipulation. We originally planned to perform gene silencing experiments using the myoblastic cell line C2C12; however, the expression level of *Lbx1* transcripts was barely detectable in these cell lines. Furthermore, we also found that even after introducing the LBX1-Myc expression vectors, these cells expressed very low levels of LBX1 protein compared to NIH3T3, possibly due to the low efficiency of lipofection gene transfer. Therefore, we decided that it was not practical to use C2C12 for our experiment. We would also like to point out that C2C12 cells are not a muscle cell line, but a myoblastic-like cell line, and do not express skeletal muscle-related genes in their undifferentiated state. In this respect, even C2C12 (or any myoblastic cell line) may not be a reasonable substitute for an in vivo model of muscle-specific *Lbx1* manipulation. We added the following in the Discussion as a limitation of the present study (P. 22); “In addition, due to technical difficulties, we performed gain-of-function experiments using the NIH/3T3 fibroblast cell line as an in vitro model, and therefore the results of the cell-based assays in this study may require further validation”.**

4. Selection of the genes linked to the energy metabolism is not clear. E.g. Bdh1 (larger fold change than Grb14) is surely involved in metabolism of energy substrates as well.

**Response: The genes selected for analysis in this study (*Mss51*, *Igfn1*, *Myh7,* and *Grb14*) were those with potential function in energy metabolism and whose increased or decreased expression in skeletal muscle was validated by quantitative PCR. *Bdh1* was indeed one of the candidate genes in the first screening, but the difference in its expression level between *LbxΔMus* and Ctrl did not reach statistical significance, and therefore excluded from further analysis. To avoid misunderstanding, we have added the following in the figure legend for S1 Fig as follows: “Black dots indicate genes with a potential role in energy metabolism whose increased or decreased expression in skeletal muscle was validated by quantitative PCR”. We have also revised the text as follows (p. 16): “We performed quantitative PCR and validated the increased or decreased expression of these genes in the skeletal muscle of *Lbx1^ΔMus^* mice and identified *Mss51*, *Igfn1*, *Myh7*, and *Grb14* as candidate genes involved in the increased energy metabolism in *Lbx1^ΔMus^* mice (Fig 4A)”.**

Minor comments: Authors should make sure that all abbreviations (e.g. AUC, CSA, ACTB) are appropriately explained. Readers would benefit from brief method description of e.g. glucose uptake analysis, cell confluence etc. (besides pointing to manufacturers instructions).

**Response: To comply with the reviewer’s comments, we have revised the text as follows:**

**A brief explanation of CSA (P. 7):**

**The cross-sectional area of adipocytes, which reflects the degree of intracellular fat accumulation, was measured using Image J software (National Institutes of Health, Bethesda, MA).**

**A brief explanation of AUC (P. 14):**

**Evaluation of the area under the curve, which reflects the total increase in blood glucose during a glucose tolerance test, showed a similar result (Fig 3B).**

**A brief explanation of ACTB (P. 33):**

**Beta-actin (ACTB) is used as an internal control.**

**Method for analyzing glucose uptake (P. 10):**

**In brief, cells (1.35 × 105 cells/well) were cultured in 2 ml of maintenance medium (DMEM supplemented with 10%fetal bovine serum and penicillin/streptomycin) in 12-well plates at 37°C. After 16 h of culture, the media were collected and centrifuged at 1,500 rpm for 5 min. The supernatants were incubated with the reaction buffers provided by the manufacturer for 30 min at 37°C, and the absorbance of the supernatant was measured at 450 nm. Glucose consumption was determined by subtracting the pre-culture glucose concentration from the post-culture glucose concentration.**

Reviewer #2:

1) The title does not seem to align with the experimental evidence presented in the work: there is not enough evidence to claim that the ablation of LBX1 "renders resistance to obesity in mice". The data presented to justify this condition are limited to a reduced weight gain in LBX1Δmus mice when subjected to a high-fat diet (HFD). Basic parameters associated with obesity, such as the Lee index, blood lipid and insulin levels, or insulin tolerance in HFD-fed animals, which would more concretely support a condition of "resistance to obesity", were not done. All other parameters analyzed (i.e., perigonadal fat, tibialis muscle weight, food intake, core temperature, and glucose tolerance) pertain to animals fed a standard diet. Additionally, the in vivo experiments on LBX1 ablation and the in vitro experiments on LBX1 overexpression indicate that altering LBX1 expression impacts glucose uptake and the expression of specific genes related to energy metabolism (particularly glucose metabolism). Together with the other in vivo evidence reported, a title more aligned with the obtained results could be: "The alteration of LBX1 expression is associated with changes in parameters related to energy metabolism"

**Response: To address the reviewer's comment, the title has been changed to read as follows: "The alteration of LBX1 expression is associated with changes in parameters related to energy metabolism in mice".**

2) In the materials and methods section, it is reported that the HFD treatment begins at 5 weeks. Why, in figure 1, are the weights of the animals shown only starting from 11 or 12 weeks? Additionally, since the duration of the HFD treatment is not indicated in the methods, it is inferred from figure 1C that this treatment lasts for 45 weeks. Why subject the animals to an HFD regime for such a long time? From an animal welfare perspective, the authors should provide adequate experimental justifications, especially considering that no additional experimental procedures other than weighing the animals are performed. In addition, the image presented in figure 1B (11-week-old regular chow-fed females as per the corresponding legend) is not consistent with the data reported in figure 1A (right panel). Such a noticeable difference in body size (1B) is not consistent with an estimated weight difference of 2 or 3 grams (1A). The authors need to better explain this discrepancy.

**Response: To address the issue raised by the reviewer, we have revised Figure 1C to show the body weight of mice starting at 5 weeks of age. We originally planned to use mice at 45-50 weeks of age, assuming that the difference in energy metabolism between Ctrl and *LbxΔMus* mice might increase at this age; however, since we did not observe a meaningful difference from the data collected from 45-week-old mice in our preliminary analysis, we decided not to include these data. Regarding animal welfare on feeding high-fat diet, we checked the “GUIDE FOR THE CARE AND USE OF LABORATORY ANIMALS (Eighth Edition)” published by the National Academy of Science; however, we were unable to find any content pertaining to this issue. Most importantly, the protocol had been approved by the Animal Care Committee at our facility and we did not observe any increase in mortality or difficulty in locomotion in the mice fed the high-fat diet during through the course of this project. Taking all of this into account, we could not find any concrete evidence to support the idea that feeding the high-fat diet to mice for 50 weeks was scientifically unethical. Needless to say, we appreciate the reviewer for reminding us of the importance of animal welfare issues when conducting experiments such as ours. With regard to the images shown in Figure 1B, we found that we mistakenly described these mice as 11 weeks old when they were both 30 weeks old. We have corrected this error in the revised manuscript. We apologize for this error and thank the reviewer for pointing it out.**

3) The authors should explain how WT females subjected to an HFD reach a body weight similar to, if not higher than, that of males, when it is known that female mice on an HFD typically achieve a lower absolute weight compared to age-matched males. This significant weight increase in WT females on HFD appears to be anomalous compared to specific literature, potentially suggesting that the genetic modification of the animal colony used in these experiments (Lbx1flox/flox) may not fully represent certain biometric parameters, especially concerning females. A comment from the authors on this issue is expected.

**Response: There was one wildtype male mouse that was smaller than the others. If we had excluded this mouse from the analysis, the average weight of the male mice would have been higher than the data presented in the study. This is the most likely reason why the average weight of the female mice appeared comparable to that of the male mice. However, since we did not pre-determine the exclusion criteria for the subjects and did not want to arbitrarily manipulate the data, we included all subjects in this experiment.**

4) Calculating the respiratory quotient (CO2 produced / O2 consumed), in addition to the VO2 value of oxygen consumed, is a metabolic parameter that, based on the study's framework and reported results, seems necessary to better define the type of metabolism in LBX1 Δmus animals (preferentially lipid, preferentially glucose, or balanced between the two).

**Response: As requested by the reviewer, we have included figures showing O2 consumption and respiratory quotient (Figures 3E and 3F). We found that O2 consumption was comparable or even higher (note that the difference did not reach statistical significance) in *LbxΔMus* mice compared to Ctrl (especially during the night), even though activity is lower in *LbxΔMus* mice compared to Ctrl (Figure 3H). On the other hand, we did not observe any difference in the respiratory quotient between Ctrl and *LbxΔMus* mice.**

5) In the materials and methods section, the method for relative quantification of RT-PCR experiments should be specified (2 –ΔΔCT ? ). In a context where authors highlight alterations in energy metabolism, how appropriate is the use of a metabolic enzyme (such as GAPDH) as a normalizer in RT-PCR experiments? Evidence (as supplementary data) should be provided to demonstrate that GAPDH expression remains unchanged in WT and LBX1 Δmus mice.

**Response: We agree with the reviewer that choosing the right internal control is always an issue in quantitative RT-PCR experiments. To address the reviewer's concern, we compared the Ct (threshold cycles) values of *Gapdh* in skeletal muscle between WT and LBX1Δmus mice and found that there was no significant difference and decided that the expression levels of *Gapdh* transcripts in skeletal muscle between WT and *LBX1Δmus* mice were comparable (S2 Fig) and could therefore be used as an internal control.**

6) In the discussion, the authors describe Mss1 as a muscle-specific transcription factor. Based on this, how confident can be considered the results of in vitro RT-PCR where LBX1 overexpression reduces Mss1 expression by over 50% compared to control cells (NIH-3T3), where this gene is not expected to be transcribed? To what extent can this consideration be extended to the other genes analyzed in these experiments? The authors should provide data supporting the actual expression of the four genes analyzed in NIH-3T3 cells.

**Response: We thank the reviewer for bringing this issue to our attention. Because *Mss51* was described as a "muscle-specific gene" in a previous study (Fujita R, et al. 2018. FASEB J 32:5012-5025), we took this for granted and did not verify the validity of this statement ourselves. On the Mouse Genome Informatics website (https://www.informatics.jax.org/), we found that transcripts for *Mss51* are expressed rather ubiquitously in mice, including the cardiovascular system, connective tissue, nervous system, and respiratory system, with no particular tissue or organ expressing high levels of *Mss51*. In light of these data, we have decided that it is misleading to describe *Mss51* as “a muscle-specific gene” and have removed this phrase from the revised text (P. 19).**

**Regarding the expression of *Mss51* transcripts and the transcripts of the other three genes (*Igfn1*, *Myh7*, and *Grb14*) in NIH-3T3 cells, we checked the Ct value of each gene examined in this study. As shown in S3 Fig, we found that the Ct value of each gene was within a reasonable range (please note that the higher the Ct value of a given gene, the lower its expression level). Although direct comparisons between different genes cannot be made by qPCR, we found that the Ct value for *Mss51* was comparable with that of *Gapdh* in NIH-3T3 cells, indicating that the expression of *Mss51* transcripts in NIH-3T3 cells was not negligible.**

7) A more thorough introduction of LBX1 would help the reader better understand the connections between LBX1, AIS, and the hypothesis that the authors aim to investigate. Specifically, are there data regarding LBX1 expression levels in AIS patients compared to healthy individuals?

**Response: As suggested by the reviewer, we have added the following to make our hypothesis easier to understand (p. 4): [6-10]. “Since the prevalence of eating disorders is lower in AIS patients than in control subjects, several studies have suggested that metabolic abnormalities, and the resulting lean physique, are potentially associated with the pathogenesis of AIS”. Regarding the expression level of LBX1 in AIS patients, to the best of our knowledge, there are almost no studies addressing this issue *. Considering that it is not feasible to collect paravertebral muscle samples from healthy children and adolescents, this may not be surprising and may be one of the reasons hindering studies in AIS.**

***** **As far as we can find in the literature, there is one study that looked at the expression level of LBX1 in patients with AIS, congenital scoliosis and lumbar disc herniation (note that healthy subjects were not included in this study). The data suggest that there is an asymmetric expression of *LBX1* in AIS patients and that the expression of *LBX1* was significantly lower in the concave side of AIS patients compared to the concave side of CS patients; however, the data are inconclusive, at least in our opinion.**

**Xu L, Dai Z, Xia C, Wu Z, Feng Z, Sun X, et al. Asymmetric Expression of Wnt/B-catenin Pathway in AIS: Primary or Secondary to the Curve? Spine (Phila Pa 1976). 2020;45(12):E677-E83. Epub 2020/02/12. doi: 10.1097/BRS.0000000000003409. PMID: 32044811.**
