# Supplementary material for: The alteration of LBX1 expression is associated with changes in parameters related to energy metabolism in mice
Source: PLoS One. 2024 Aug 7;19(8):e0308445. doi: 10.1371/journal.pone.0308445 (PMC11305531; doi:10.1371/journal.pone.0308445)
Supplement: Attachment — Submitted filename: responses.docx [file pone.0308445.s007.docx]

**Point-by-point responses**

Reviewer #1:

1. Authors addressed all the comments and included additional data (mostly as Supplementary figures). However, the supplementary figures are not commented in the main text. S4 Figure is not even included in the List of Supporting information.

**Response: We apologize for inadvertently omitting S4 Fig from the list of supporting information. We have added it to the revised manuscript. Regarding the citation of supplementary materials in the text, the PLOS ONE instructions indicate that they do not need to be cited in the text (see below).**

**“We recommend that you cite supporting information in the manuscript text, but this is not a requirement. Cite the files using the format outlined in Item Description. If you cite supporting information in the text, citations do not need to be in numerical order.”**

**https://journals.plos.org/plosone/s/supporting-information#loc-item-description**

2. The new data (mean energy expenditure, respiratory quotient) rather do not support the original hypothesis that limited weight gain is due to increased energy expenditure. These parameters do not show significant differences and the tendencies can be completely reverted by different way of normalization (per mouse vs per body weight) as discussed in Author´s response but not in the new version of manuscript. If these limitations are properly disclosed, the reader can make more balanced conclusion based on the presented data.

**Response: To address the reviewer’s concern, we have included the following as a limitation in the Discussion section (p. 22): “Second, total body weight, but not lean body weight, was used to normalize the VO_2_ max and O_2_ consumption values (Figs 3C and 3E). We found that the differences in basal O_2_ consumption between Ctrl and *Lbx1^ΔMus^* mice were abolished when unadjusted by body weight (S4 Fig). However, considering that *Lbx1^ΔMus^* mice have underdeveloped forelimbs and therefore less muscle than Ctrl mice, that there is no marked difference in fat weight at the time of analysis (11 weeks of age) (Fig 2A), that total activity is significantly lower in *Lbx1^ΔMus^* mice than in Ctrl mice (Fig. 3H), and that there is no difference in food intake (Fig 3G), it is conceivable that total energy expenditure is higher in *Lbx1^ΔMus^* mice than in Ctrl mice.”**

**Regarding the respiratory quotient, our understanding is that it does not reflect the level of total energy expenditure, but only the ratio of energy pathways used for fats, carbohydrates, and proteins. Therefore, even if there were no differences in respiratory quotient values between Ctrl and *Lbx1^ΔMus^* mice, we cannot conclude that there was no difference in energy expenditure.**

Authors also clarify that genes in focus were selected based on "a potential role in energy metabolism" and differences in expression "validated by quantitative PCR". It would imply that several more (or even all of?) genes from Figure S1 were checked by qPCR, but only few of them were successfully validated. If this is the case, than the list of primers should include primers for all the genes tested and not only those successfully validated.

**Response: We have included the list of genes we examined in Table 1. We have also added a brief comment in the Figure legend (S1 Fig) as follows: “The transcriptional expression levels of *Bdh1, Igfbp5, Irs2, Nr4a2*, and *Nr4a3* were also examined, but the difference in expression could not be reproduced by quantitative PCR.”**

Reviewer #2: The authors have satisfactorily responded to all my questions and made the necessary changes to the manuscript.

**Response: We are pleased to learn that the concerns raised by the reviewer have been properly addressed. We would like to thank the reviewer again for her/his constructive comments.**
